# Do Proton Pump Inhibitors Decrease Calcium Absorption?

**DOI:** 10.1002/jbmr.166

**Published:** 2010-06-24

**Authors:** Karen E Hansen, Andrea N Jones, Mary J Lindstrom, Lisa A Davis, Toni E Ziegler, Kristina L Penniston, Amy L Alvig, Martin M Shafer

**Affiliations:** 1 Departments of Medicine, University of Wisconsin Madison, WI, USA; 2 Departments of Biostatistics and Medical Informatics, and University of Wisconsin Madison, WI, USA; 3 Center for Translational and Clinical Research Core; and the University of Wisconsin Madison, WI, USA; 4 National Primate Research Center, University of Wisconsin Madison, WI, USA; 5 Departments of Urology; the University of Wisconsin Madison, WI, USA; 6 Wisconsin State Lab of Hygiene Madison, WI, USA

**Keywords:** ACID SUPPRESSANT, BONE, CALCIUM ABSORPTION, FRACTURE, OSTEOPOROSIS

## Abstract

Proton pump inhibitors (PPIs) increase osteoporotic fracture risk presumably via hypochlorhydria and consequent reduced fractional calcium absorption (FCA). Existing studies provide conflicting information regarding the direct effects of PPIs on FCA. We evaluated the effect of PPI therapy on FCA. We recruited women at least 5 years past menopause who were not taking acid suppressants. Participants underwent three 24-hour inpatient FCA studies using the dual stable isotope method. Two FCA studies were performed 1 month apart to establish baseline calcium absorption. The third study occurred after taking omeprazole (40 mg/day) for 30 days. Each participant consumed the same foods during all FCA studies; study meals replicated subjects' dietary habits based on 7-day diet diaries. Twenty-one postmenopausal women ages 58 ± 7 years (mean ± SD) completed all study visits. Seventeen women were white, and 2 each were black and Hispanic. FCA (mean ± SD) was 20% ± 10% at visit 1, 18% ± 10% at visit 2, and 23% ± 10% following 30 ± 3 days of daily omeprazole (*p* = .07, ANOVA). Multiple linear regression revealed that age, gastric pH, serum omeprazole levels, adherence to omeprazole, and 25-hydroxyvitamin D levels were unrelated to changes in FCA between study visits 2 and 3. The 1,25-dihydroxyvitamin D_3_ level at visit 2 was the only variable (*p* = .049) associated with the change in FCA between visits 2 and 3. PPI-associated hypochlorhydria does not decrease FCA following 30 days of continuous use. Future studies should focus on identifying mechanisms by which PPIs increase the risk of osteoporotic fracture. © 2010 American Society for Bone and Mineral Research.

## Introduction

Proton pump inhibitors (PPIs) were identified recently as an independent risk factor for osteoporotic fracture.(1–6) Millions of American adults take PPIs to treat or prevent gastrointestinal conditions.(7) Indeed, PPIs rank second only to statins in drug sales.(8) Moreover, postmenopausal women account for over half of long-term PPI users(8) and represent the subset of individuals at greatest risk for osteoporotic fracture. Therefore, this newly identified fracture risk factor has important public health ramifications.

Three large case-control studies(1–3) and one retrospective cohort study(4) reported that PPI therapy increased the relative risk of fracture by 1.15 [95% confidence interval (CI) 1.10–1.20](4) to 1.92 (95% CI 1.16–3.18).(3) Subsequently, three prospective studies designed to identify risk factors for osteoporotic fracture also found that PPI therapy was an independent risk factor for fracture.(5,6) Among 5339 women enrolled in the Study for Osteoporotic Fractures, those reporting PPI use at the sixth visit (*n* = 234) had a higher risk of nonvertebral fracture (relative hazard = 1.34, 95% CI 1.10–1.64) during an average follow-up of 5 years.(5) Among 5775 men enrolled in the Osteoporotic Fractures in Men (MrOS) study, PPI use was associated with a higher risk of fracture, but only in men not taking calcium supplements (relative hazard = 1.49, 95% CI 1.04–2.14).(5) In a third prospective study, 5% of 1211 women were taking omeprazole at study entry.(6) Omeprazole therapy was an independent risk factor for vertebral fracture (relative risk = 3.50, 95% CI 1.14–8.44) during 6 years of follow-up.(6)

PPIs hypothetically increase the risk of osteoporotic fracture by causing hypochlorhydria, reduced intestinal calcium absorption, and subsequent negative calcium balance.(5,7) Since calcium solubility depends on the pH of the solution, calcium absorption likewise may depend on gastric pH. This supposition was supported by a study(9) of 11 achlorhydric subjects who in the fasting state demonstrated impaired absorption of a calcium carbonate gelatin capsule but normal absorption of a calcium citrate solution. However, absorption of calcium carbonate was restored to normal when these subjects consumed calcium carbonate capsules with breakfast.(9) Typically, individuals ingest calcium from dietary sources instead of supplements. Thus the ability of achlorhydric patients to absorb calcium with a meal would seem more applicable to the ability to absorb calcium while taking PPIs.

Other studies cast significant doubt on the import of gastric acid and calcium solubility on subsequent calcium absorption. In one study,(10) calcium carbonate was administered with a meal to eight adults. Absorption of calcium carbonate was measured twice by lavage, once when subjects' gastric pH was maintained at 7.4 using NaHCO_3_ infusion and again when gastric pH was maintained at 3.0 using HCl infusion.(10) Calcium absorption was identical in both conditions despite a marked difference in calcium carbonate solubility at differing pH.(10) Subsequently, another group performed a post hoc analysis of calcium absorption data collected from 352 subjects across multiple studies using calcium sources that varied in solubility by five orders of magnitude.(11) The relationship between calcium solubility and absorption was weak; calcium absorption was more strongly related to food components coingested with the calcium salt.(11)

Five studies(12–16) (Table 1) have investigated the direct effect of PPIs on intestinal calcium absorption with discordant results. However, important limitations of these studies prevent definitive conclusions regarding the effect of PPIs on calcium absorption. First, none of the studies used dual isotopes to measure calcium absorption, and three used serum calcium levels, which correlate only weakly(17) with absorption data obtained using the “gold standard” dual-isotope method. Second, the duration of PPI therapy was less than 12 days in four studies. The Institute of Medicine recommends that researchers wait 12 days for equilibrium to occur before measuring changes in calcium homeostasis following an intervention.(18) Third, one study(13) measured calcium absorption in the fasting state, which might not reflect calcium absorption efficiency with a meal. Finally, one study(12) did not measure calcium absorption prior to the onset of PPI therapy. It therefore remains uncertain whether PPI-associated hypochlorhydria truly decreases calcium absorption.

**Table 1:** Studies Assessing Changes in Calcium Absorption Related to PPI Therapy

Study	Subjects	Intervention	Calcium absorption methodology	Result
Serfaty-Lacrosniere, 1995(12)	13 healthy adults, median age 59 years	Omeprazole 40 mg daily for 7 days	Calcium consumed with a meal, absorption determined by intestinal lavage	No difference in calcium absorption between treatment groups; calcium absorption not altered in all subjects following gastric infusion of 120 mL 0.1 M hydrochloric acid
Graziani, 1995(14)	8 healthy men, mean age 38 years	Baseline and again after omeprazole 20 mg every 8 hours for 3 days	Postprandial increment in serum calcium	Lack of increase in serum calcium with omeprazole therapy, suggesting decreased calcium absorption
Hardy, 1998(16)	16 dialysis patients, mean age 61 years	Baseline and again after omeprazole 20 mg daily for 2 months	Serum calcium measured weekly at beginning of dialysis	Lower serum calcium during omeprazole therapy, suggesting decreased calcium absorption
Graziani, 2002(15)	30 dialysis patients, mean age 57 years	Baseline and again after omeprazole 20 mg every 8 hours for 3 days	Postprandial increment in serum calcium	Lack of increase in serum calcium with omeprazole therapy, suggesting decreased calcium absorption
O'Connell, 2005(13)	Postmenopausal women, mean age 76 years	Omeprazole 20 mg daily for 7 days and placebo daily for 7 days	Fasting serum ^45^Ca isotope level 5 hours after consuming 500 mg ^45^Ca carbonate	Calcium absorption decreased from 9% to 4% following omeprazole therapy (*p* < .05)

We designed a prospective study using dual stable calcium isotopes to evaluate changes in fractional calcium absorption among postmenopausal women related to omeprazole therapy.

## Materials and Methods

### Patient population

Women at least 5 years past the onset of menopause, defined as the date of last menses, were eligible for participation. We excluded women reporting an allergy to PPI, those taking prescription medication for heartburn (PPI or H_2_-blocker therapy), and those taking over-the-counter antacids within the past 2 months. Women with intestinal conditions associated with malabsorption or hypochlorhydria, stage 4 or 5 chronic kidney disease, or nasal anatomy preventing nasogastric tube placement also were excluded. We further barred women taking medications known to alter calcium metabolism or interact with omeprazole. The University of Wisconsin (UW) Human Subjects Committee reviewed and approved the study protocol, and the study was registered as a clinical trial (ClinicalTrials.gov identifier: NCT00582972).

We recruited subjects through e-mail letters of invitation and screened women who called our center via phone. Interested and potentially eligible subjects underwent a screening visit to confirm eligibility and provide written informed consent. We recruited women of all races and ethnic groups. Race was assessed and recorded at the screening visit because African Americans absorb calcium more efficiently than white people.(19)

Prior to the first calcium absorption study, subjects completed 7-day food diaries using a food scale. The study nutritionist (LAD) used Food Processor software (ESHA Research, Salem, OR, USA) to assess subjects' energy, macronutrient, fiber, calcium, iron, magnesium, sodium, vitamin D, oxalate, and caffeine intake. We replicated each subject's typical intake of all nutrients during each of her 25-hour hospital stays; her breakfast, lunch, dinner, and snacks were identical at each of the three study visits. Food not consumed during each stay was bagged and weighed. Subsequently, the study nutritionist analyzed subjects' intake of nutrients during each research admission. During the study, subjects continued current nutritional supplement use to ensure that changes in calcium absorption were unrelated to changes in calcium intake. Throughout the study, investigators tracked any changes in supplement use, including calcium supplements.

### Study interventions

We measured participants' intestinal calcium absorption on three separate occasions using the dual stable calcium isotope protocol.(20) Stable isotopes are calcium atoms of different atomic weights with no radioactivity.(21) The dual-isotope method is considered the “gold standard” method by which to measure calcium absorption(21) owing to its accuracy, lack of toxicity,(21) and essentially identical biochemical behavior(21) to that of the dominant calcium atom, ^40^Ca. We administered one isotope (^44^Ca) orally to trace fractional calcium absorption (FCA) and the other isotope (^42^Ca) intravenously to trace renal and intestinal calcium recycling. We used a formula(20) incorporating the isotope doses and their concentrations in participants' 24-hour urine collections to calculate FCA.

Trace Sciences International (Wilmington, DE, USA) provided ^42^Ca and ^44^Ca as calcium carbonate powder. The UW Waisman Clinical Biomanufacturing Facility reconstituted isotopes into calcium chloride solutions using the NIH protocol, including tests for sterility and pyrogenicity.(22) Once cleared for human use, the isotope solutions were stored at the UW Pharmacy Research Center until administration to subjects.

For all 24-hour inpatient FCA studies, subjects fasted after midnight and arrived at the UW Center for Translational and Clinical Research Core (CTRC) at 7:00 a.m. On arriving on the ward, subjects emptied their bladder to provide a second voided urine sample for measurement of C-telopeptide, a marker of bone resorption. The 24-hour urine collection began immediately after this void. Subsequently, an intravenous line was placed and blood collected to measure parameters associated with calcium homeostasis, including serum calcium, creatinine, 25-hydroxyvitamin D [25(OH)D], 1,25-dihydroxyvitamin D_3_ [1,25(OH)_2_D_3_], and intact parathyroid hormone (PTH).

During breakfast, subjects drank a small volume [50 ± 7 mL (mean ± SD)] of Tropicana Pure Premium orange juice fortified with calcium citrate malate and vitamin D (pH 4.3 ± 0.1)(23) and mixed with approximately 8 mg/16 mL of ^44^Ca chloride solution. Simultaneously, subjects received approximately 3 mg/6 mL of ^42^Ca intravenously followed by approximately 100 mL of normal saline. Nurses weighed isotope vials in duplicate before and after use to record the administered doses of ^42^Ca and ^44^Ca. Subsequently, nurses supervised complete inpatient 24-hour urine collections and recorded subjects' nutrient intake during each stay. We calculated that 50 mL of Tropicana orange juice at a pH of 4.3(23) contains 0.003 mmol of H^+^ ions. During breakfast, humans secrete 37 ± 4 mmol of H^+^ ions over 2 hours.(24) Thus 50 mL of orange juice does not significantly affect gastric pH.

We performed two baseline absorption studies to evaluate the monthly variability in FCA without intervention. The morning prior to discharge during the first (*n* = 10) or second (*n* = 8) study, we measured subjects' fasting gastric pH via nasogastric tube.(25) We scheduled the third FCA study to occur on the thirtieth day of omeprazole therapy. Subjects received a supply of 20-mg omeprazole tablets with instructions to take two tablets at least ½ hour before breakfast every day. The third study was identical to the first two, with the additional measurement of morning serum omeprazole levels and pill counts to assess adherence.

We chose a 30-day interval between subjects' calcium absorption study visits for two reasons. First, based on sequential analysis of urine isotope levels 5 days following dosing in another study,(22) we estimated that the half-life of calcium isotopes was approximately 21 hours. Thus a 30-day interval between study visits ensured that no residual isotope remained from the prior study to spuriously elevate absorption measurements in the next study. Second, the inhibitory effect of once-daily omeprazole on gastric acid secretion plateaus after approximately 4 days (package insert). The Institute of Medicine recommends that researchers wait 12 days for equilibrium to occur before measuring changes in calcium homeostasis following an intervention.(18) We therefore decided to extend the interval between study visits to 30 days in consideration of these issues.

### Laboratory analysis

The UW Hospital and Clinics Laboratory (Madison, WI, USA) measured serum chemistries and intact PTH and 25(OH)D levels. Personnel measured PTH using an electrochemiluminescence immunoassay kit with reported intra- and interassay coefficients of variation (CVs) of 4.3% and 3.3%, respectively. 25(OH)D levels were measured using a semiautomated solid-phase-extraction reverse-phase HPLC assay with intra- and interassay CVs of 4.2% and 5.0%, respectively. Associated Regional University Pathologists (Salt Lake City, Utah, USA) measured 1,25(OH)_2_D_3_ using a radioimmunoassay kit with intra- and interassay CVs of 7.7% and 12.3%, respectively. The UW National Primate Center Assay Services Laboratory (Madison, WI, USA) measured urine C-telopeptide (CTX) using the β-crosslaps ELISA kit from ImmunoDiagnostics Systems (Bolden, UK) with intra- and interassay CVs of 1.0% and 10.9%, respectively.

Omeprazole levels were measured at the Primate Center Laboratory following the methods of Zarghi and colleagues.(26) We thawed plasma samples and mixed 500 µL of each sample with 50 µL of carbamazepine (internal standard 2 µg/mL), 500 µL of acetonitrile, and 100 µL of saturated sodium chloride solution. Samples were centrifuged for 15 minutes at 8000 rpm, and 40 µL of supernatant was injected onto an HPLC (Beckman Instruments, System Gold, consisting of No. 126 solvent module, a No. 168 diode array detector, and a No. 508 autosampler) equipped with a Chromolith Performance RP-18 endcapped 4.6 × 100 mm separation column (Merck, Darmstadt, Germany). Retention times were (mean ± SE) 3.5 ± 1.35 minutes for omeprazole and 6.0 ± 3.07 minutes for carbamazepine as internal standards. Standards (*n* = 7) were run at 3.0 to 250 ng/40 µL injection. All samples were analyzed in duplicate in one single run; the intraassay CV was 1.43%.

We measured the calcium concentrations and isotope ratios of the urine samples at the Wisconsin State Lab of Hygiene using high-resolution inductively coupled plasma mass spectrometry (HR-ICP-MS; Finnigan Element 2, Thermo Instruments, Bremen, Germany).(27) Prior to analysis, we isolated calcium from urine by oxalate precipitation.(22) We diluted specimens to a total calcium level of 6 ± 1 mg/L. Samples were introduced into the HR-ICP-MS using a high-precision quartz cyclonic spray chamber and Teflon medium-flow (400 µL/min) nebulizer, and data were acquired for three target isotopes (^42^Ca, ^43^Ca, and ^44^Ca), doubly charged strontium, and scandium (internal standard). Reference standards (High Purity Standards Calcium) were placed at frequent intervals (every two samples) throughout the analytical sequence to internally normalize the data and correct for isotopic ratio drift, as well as reference ratios to a recognized external NIST-traceable standard.

We obtained data in the medium-resolution mode (4000) of the HR-ICP-MS to resolve all significant spectral interferences on the target isotopes. Under typical operating conditions, the HR-ICP-MS exhibited 0.2 × 10^6^ counts/s of ^43^Ca (3 × 10^6^ for ^44^Ca) at the target Ca concentration. Typical precision for both reference standards and urine samples was 0.2% to 0.4%. We analyzed each urine sample on at least two occasions in separate batches and used averages of data to calculate FCA. The Pearson correlation coefficient for values obtained by duplicate analyses of urine specimens was *r* = 0.98 (*p* < .0001) for ^42^Ca/^43^Ca and *r* = 0.97 (*p* < .0001) for ^44^Ca/^43^Ca.

### Study outcomes

The primary study outcome was the change in FCA following 1 month of omeprazole therapy. Secondary outcomes included the change in bone resorption (CTX) and the monthly variability in FCA at baseline, prior to omeprazole therapy.

### Statistical analysis

Sample size calculations were based on data from a subset of 5452 women enrolled in the Study for Osteoporotic Fractures, in whom an 8% lower FCA was associated with a 1.2-fold (95% CI 1.05–1.48) greater risk of hip fracture.(28) In a previous study at our center,(22) the standard deviation for FCA between postmenopausal women was 8% and within women was less than 1%. Thus a sample size of 20 women allowed us to detect a 7% or greater difference in FCA after omeprazole therapy with 90% power and a two-sided type 1 error of 0.05. We planned to recruit up to 25 women to allow for potential dropout during the study.

For calculated values, we reported the mean ± SD unless otherwise indicated. We used analysis of variance (ANOVA) with the Bonferroni correction to compare within-subject changes for FCA, bone resorption, laboratory characteristics, and dietary habits. Multiple regression was used to evaluate relationships between PPI-associated changes in FCA and subject variables, including age, basal gastric pH, adherence to omeprazole, and vitamin D metabolites [25(OH)D and 1,25(OH)_2_D_3_]. Variables reflecting adherence included percent adherence, number of omeprazole tablets taken, and serum omeprazole levels. We completed all analyses using the R statistical analysis system (Version 2.6.0).(29)

## Results

**Fig. 1:**
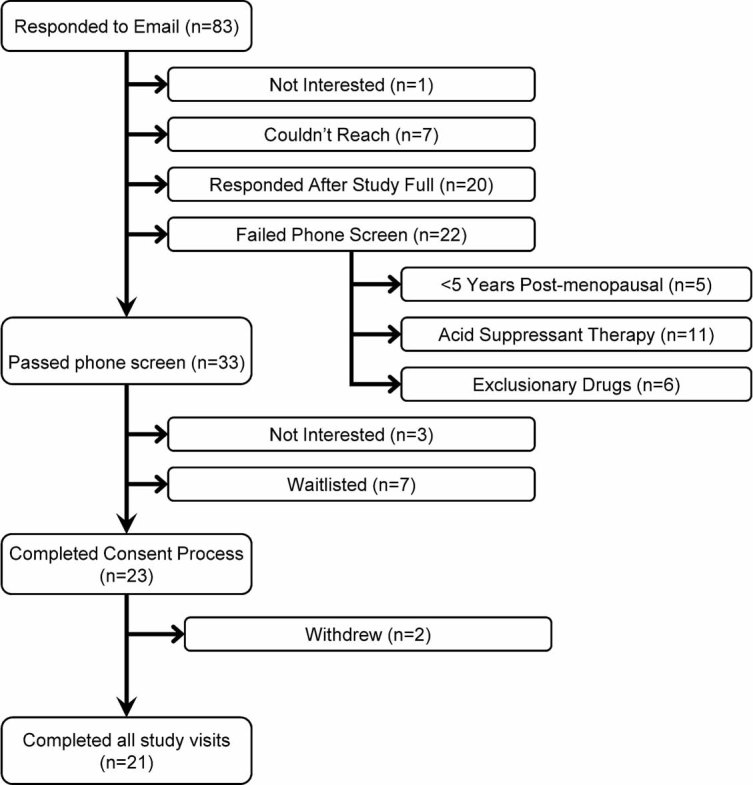
Study recruitment.

We recruited women by sending mass e-mail advertisements to UW female faculty in January 2008 (Fig. 1). Eighty-three women called our research center in response to the email, and 33 were interested and eligible on the basis of a phone interview. Twenty-three women came to the CTRC and provided written informed consent to enter the study. Two women could not complete the 7-day diet diary and withdrew from the study. Thus 21 women completed all study visits.

Table 2 summarizes subjects' characteristics (*n* = 21). Their mean age was 58 ± 7 years; 17 women were white, and 2 each were black and Hispanic. Subjects' mean 25(OH)D and PTH levels at baseline were 28 ± 13 ng/mL and 49 ± 24 pg/mL, respectively.

**Table 2:** Subjects' Anthropomorphic, Laboratory, and Dietary Characteristics

Characteristics	Screening	Study 1	Study 2	Study 3
Anthropomorphic characteristics				
Age, years	58 ± 7	—	—	—
Race	17 Caucasian	—	—	—
	2 Black			
	2 Hispanic			
Body mass index, kg/m^2^	—	29 ± 5	29 ± 5	29 ± 5
Laboratory characteristics				
25(OH)D, ng/mL	—	28 ± 13	36 ± 18	35 ± 16
1,25(OH)_2_D_3_, pg/dL	—	47 ± 26	53 ± 24	60 ±18
PTH, pg/mL	—	49 ± 24	50 ± 22	49 ± 20
Serum calcium, mg/dL	—	9.1 ± 0.4	9.2 ± 0.3	9.2 ± 0.4
Serum creatinine, mg/dL	—	0.8 ± 0.1	0.8 ± 0.1	0.8 ± 0.1
Urine calcium, mg/24 h	—	153 ± 67	152 ± 64	146 ± 46
Dietary habits				
Kilocalories^a^	2200 ± 340	2100 ± 310	2100 ± 320	2100 ± 300
Calcium, mg	1400 ± 650	1400 ± 650	1400 ± 650	1400 ± 650
Carbohydrates, g^b^	270 ± 60	250 ± 50	250 ± 50	250 ± 50
Protein, g	88 ± 21	84 ± 20	83 ± 20	83 ± 20
Fat, g	87 ± 22	85 ± 22	85 ± 23	85 ± 23
Fiber, g	24 ± 11	23 ± 10	22 ± 9	23 ± 10
Vitamin D, IU^c^	160 ± 100	150 ± 100	150 ± 100	150 ± 100
Sodium, mg^a^	3400 ± 890	3300 ± 820	3300 ± 810	3300 ± 820
Magnesium, mg	360 ± 110	360 ± 120	360 ± 110	360 ± 110
Iron, mg	16 ± 5	16 ± 6	16 ± 6	16 ± 6
Caffeine, mg	170 ± 150	160 ± 110	160 ± 110	160 ± 110
Oxalate, servings	1.2 ± 1.0	1.1 ± 1.0	1.2 ± 1	1.4 ± 1

*Note:* Fractional calcium absorption studies 1, 2, and 3 occurred at baseline, 39 ± 17 days, and 64 ± 2 days later, respectively. We used analysis of variance with a Bonferroni correction to compare within-subject changes in laboratory characteristics and dietary habits during study visits. Four dietary parameters were statistically different during inpatient visits compared with subjects' usual dietary intake based on 7-day diet diaries secondary to incomplete meal consumption.

a *p* < .0001.

b *p* = .0015.

c *p* = .005.

Through the combination of dietary and supplemental intake, subjects ingested 1400 ± 650 mg of calcium per day, approximately half (46%) consumed with breakfast. The calcium content of the breakfast varied between subjects (700 ± 600 mg); however, each woman's calcium intake from diet and supplements was identical at each of her three study visits. Twelve subjects (57%) took supplements containing calcium prior to the research study; these were continued during inpatient stays (Table 2).

Six women reported occasional heartburn, but only one woman carried a diagnosis of gastroesophageal reflux disease. No participant reported antacid use within the past 2 months. Three women had significant gag reflex preventing placement of the nasogastric tube. Baseline gastric pH was 3.4 ± 1.8 (range 0 to 6) among the remaining 18 women, similar to levels reported among women in another study.(30) Seven women had a gastric pH between 0 and 3, and 11 had a gastric pH greater than 3.

**Fig. 2:**
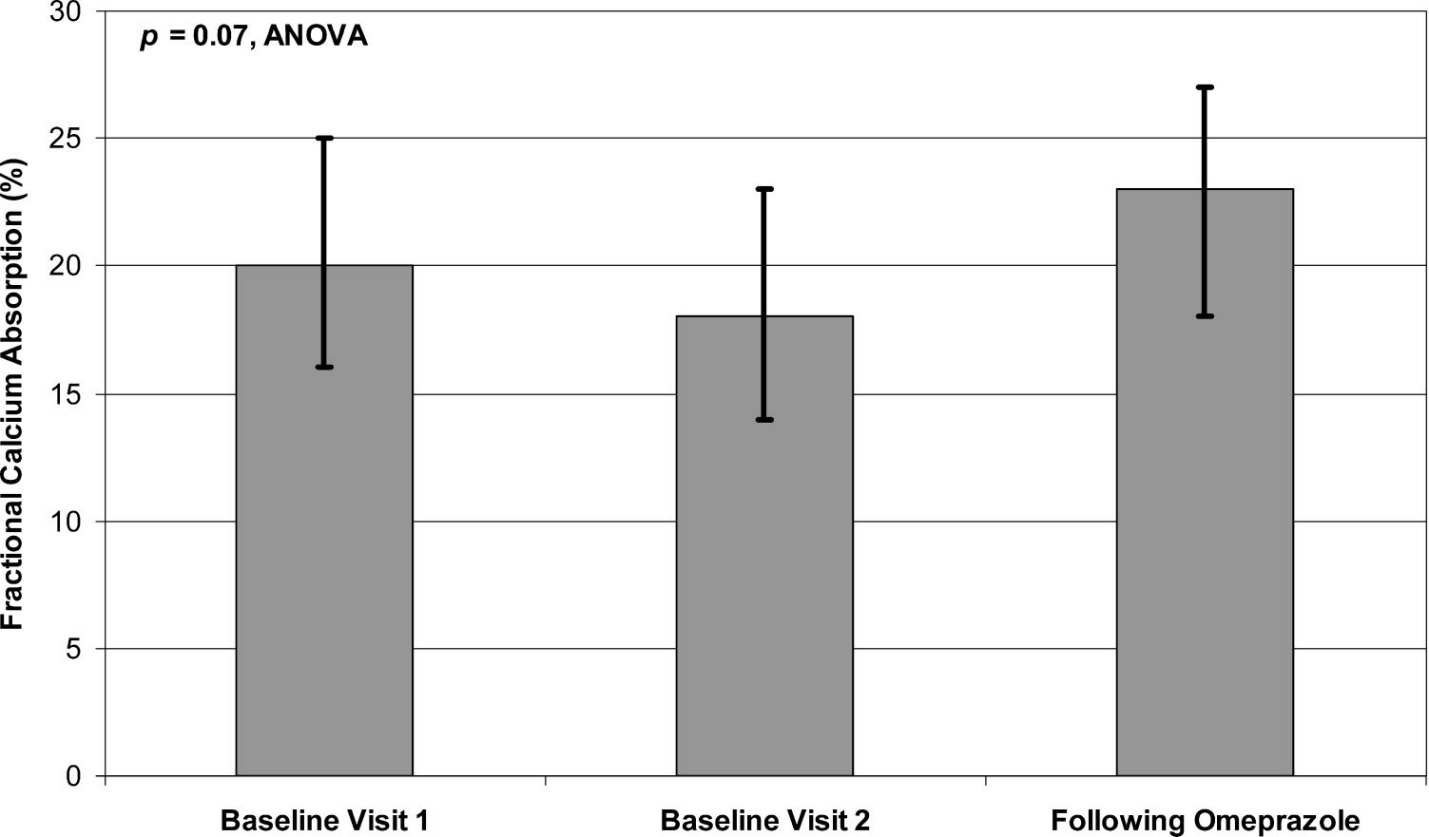
We measured fractional calcium absorption in participants (*n* = 21) on three separate occasions. Each subject completed her third calcium absorption study after taking omeprazole 40 mg daily for 30 days. The study group's mean FCA was 20% at visit one, 18% at visit two, and 23% following omeprazole therapy (*p* = .07, ANOVA). In this figure, we illustrate absorption using the means and 95% confidence intervals.

We measured subjects' FCA at baseline, 39 ± 17 days, and 64 ± 2 days later, the third visit occurring 30 ± 3 days after onset of daily omeprazole therapy. The study group's FCA (mean ± SD) was 20% ± 10% at visit one, 18% ± 10% at visit two, and 23% ± 10% following omeprazole therapy (*p* = .07, ANOVA; Fig. 2). The mean change in FCA from visit 1 to visit 3 was 2% ± 7% and from visit 2 to visit 3 was 4% ± 8%.

We performed post hoc analyses to evaluate whether subsets of subjects experienced a significant decline in FCA from visit 2 to visit 3 following omeprazole therapy. FCA increased by 3% ± 6% in 12 women taking calcium supplements and increased by 4% ± 10% in 9 women not taking calcium supplements (*p* = .78 for the difference between groups). Similarly, FCA increased by 5% ± 8% among 7 women with a gastric pH ≤ 3 and increased by 1% ± 5% among 11 women with a gastric pH > 3 (*p* = .24 for the difference between groups). A scatter plot revealed no correlation between baseline gastric pH and the change in FCA between visit 2 and visit 3 (*r* = –0.17, *p* = .52).

PTH and serum and urine calcium levels remained unchanged following omeprazole (Table 2). Multiple regression models revealed that age, serum omeprazole levels, percent adherence, number of omeprazole pills taken, gastric pH, and 25(OH)D levels were unrelated to changes in FCA associated with omeprazole therapy. The 1,25(OH)_2_D_3_ level at visit 2 was the only variable that was significantly associated with the change in FCA between visits 2 and 3 (estimate –0.002, *p* = .049).

Per protocol, participants ingested omeprazole 40 mg daily for 30 ± 3 days. Based on pill counts, adherence was excellent (mean adherence 98%, range 87% to 100%). Among participants, serum omeprazole levels were 545 ± 551 ng/mL, closely resembling levels reported in other studies.(31) Two women took their last omeprazole tablets the day prior to admission, but since omeprazole suppresses gastric acid production for more than 24 hours after dosing,(32) we judged that measurements of calcium absorption in these women would reflect the effects of omeprazole on FCA. A post hoc analysis revealed no significant PPI-induced change in FCA when excluding these 2 women from the analysis.

No woman experienced severe side effects of omeprazole warranting its discontinuation. Ten women identified mild omeprazole side effects, including nausea (*n* = 2), diarrhea (*n* = 2), constipation (*n* = 2), flatulence (*n* = 2), and headache (*n* = 1). One woman noted a sense of scratching in her throat and increased sputum production.

**Fig. 3:**
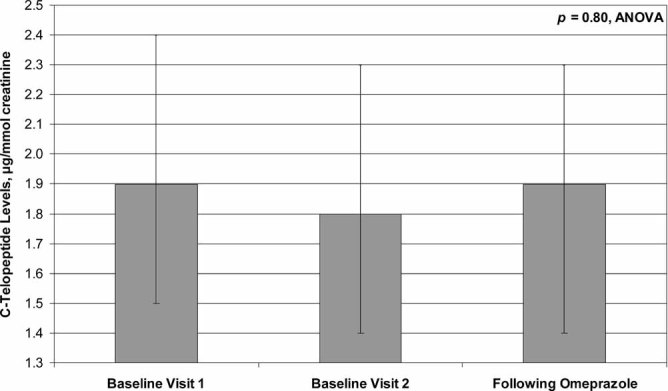
We assessed bone resorption by measuring participants' (*n* = 21) fasting morning urine CTX levels. Subjects' mean CTX values at visits 1, 2, and 3 were 1.9, 1.8, and 1.9 µg/mmol of creatinine, respectively (*p* = .80, ANOVA). In this figure we summarize CTX levels using the means and 95% confidence intervals.

In vitro studies(33) suggest that PPIs might decrease bone resorption by inhibiting the osteoclastic H^+^-ATPase pump, which might improve bone health. However, two clinical studies(34,35) report divergent results on the effect of PPIs on bone resorption in humans. Subjects' CTX levels did not change significantly during our study (*p* = .80, ANOVA; Fig. 3). Subjects' CTX values were 1.9 ± 1.0 µg/mmol of creatinine at visit one, 1.8 ± 1.0 µg/mmol of creatinine at visit two, and 1.9 ± 0.9 µg/mmol of creatinine at visit three.

We used 7-day diet diaries to replicate subjects' nutritional habits during study visits. However, participants were not required to eat 100% of meals during inpatient stays. Subsequently, 4 of 12 dietary variables were statistically different but not clinically different during inpatient stays when compared with outpatient food records (Table 2). Indeed, the within-subject SD for change in intake of kilocalories (111 kcal), carbohydrates (14 g), vitamin D (12 IU), and sodium (122 mg) represented small percentage changes in typical intakes of these four nutrients (ranging from 3.5% to 7%) that resemble daily variations in dietary intake. We also calculated the potential renal acid load (PRAL)(36) of the breakfast meal (–3.4 ± 7.9) and found no relationship between PRAL and baseline FCA.

## Discussion

Recent studies(1–6) link PPI therapy to higher rates of osteoporotic fracture, allegedly because PPI-induced hypochlorhydria decreases calcium solubility, leading to decreased intestinal calcium absorption and negative calcium balance. Although the absolute increase in risk of fracture appears small, it is important because millions of American adults take PPIs to treat or prevent gastrointestinal conditions.(7) Previous studies have failed to establish unequivocally that PPIs increase fracture risk by decreasing calcium absorption, but these studies have potential limitations, as discussed earlier. We found that 30 days of continuous PPI therapy did not decrease intestinal calcium absorption in postmenopausal women. Additionally, subjects' PTH, serum calcium, and urine calcium levels remained stable, providing further evidence that PPIs do not alter calcium absorption or calcium balance in the short term.

Our study had several strengths. We employed a “gold standard” technique to measure FCA(20) and conducted studies on the inpatient ward, permitting complete 24-hour urine collections. We replicated subjects' nutritional habits during studies in order to carefully assess the effect of PPI therapy on FCA, independent of alterations in nutritional practices, including calcium intake. We performed two FCA studies at baseline in all participants, thus controlling for age, dietary habits, and other variables that can influence FCA independent of PPI therapy.(37) Adherence to omeprazole was excellent based on pill counts. We used a prescription dose of omeprazole, permitting application of study findings to other prescription-strength PPIs. The high precision of our calcium absorption study method permitted the ability to detect small changes in FCA. Among 21 subjects, the standard deviation for the monthly change in FCA between visits 1 and 2 was 8.2%. Thus we had 80% power to detect a 5.3% or greater change in FCA following omeprazole and 75% power to detect a 1% or greater change in FCA.

The greatest limitation of our study was its short-term nature. The Institute of Medicine recommends that researchers wait 12 days for equilibrium to occur before measuring changes in calcium homeostasis following an intervention.(18) We extended our treatment interval to 30 days, but this might be insufficient to detect true long-term changes in FCA from PPIs. Notably, many patients take PPIs for years. A prospective intervention study of longer duration should be performed to confirm the findings of this study. Additionally, a study assessing changes in calcium absorption following cessation of PPIs might be informative, although rebound reflux symptoms might interfere with successful ability to stop PPI therapy.(38) Two participants took their last dose of omeprazole the day prior to admission, but a post hoc analysis indicated no differential change in FCA when excluding these women. Additionally, omeprazole's effect on gastric acid production persists for more than 24 hours after dosing,(32) with gradual return of gastric acidity 3 to 5 days after cessation of therapy (package insert). We did not measure gastric pH following omeprazole therapy and therefore cannot determine whether women with greater changes in gastric pH may have experienced a decrease in calcium absorption following omeprazole therapy. Our participants were relatively young and healthy; PPIs might decrease calcium absorption in elderly adults or those with multiple comorbidities. Finally, PPI therapy simply might mark people with impaired absorption of nutrients owing to their underlying gastrointestinal illnesses; we excluded such subjects from this study.

Subjects' average 25(OH)D level indicated insufficiency during the first calcium absorption study visit and sufficiency at subsequent visits. However, we found no statistical differences in subjects' serum 25(OH)D levels throughout the study when analyzing serum 25(OH)D as a continuous variable. Multivariate analyses revealed that 25(OH)D was not a significant variable affecting fractional calcium absorption. Finally, we would not expect small changes in serum 25(OH)D levels to influence fractional calcium absorption in a meaningful way based on our previous publication,(22) in which very large increments in serum 25(OH)D yielded only small increases in calcium absorption. Indeed, using the dual stable calcium isotope technique, we documented that calcium absorption increased by only 3% ± 1% (*p* = .04) when subjects' serum vitamin D levels increased from 22 ± 4 to 64 ± 21 ng/mL following prescription of high-dose vitamin D.

It seems simplistic to believe that intestinal calcium absorption, 90% of which occurs in the small intestine, depends on gastric acidity. While the stomach secretes 2500 mL/day to intestinal contents, these acidic secretions are neutralized by approximately 3500 mL of alkaline secretions, including saliva (∼1500 mL), bile (500 mL), and pancreatic juices (1500 mL).(39) As a result, the pH of contents in the upper duodenum is typically between 6.0 and 7.0.(39) Thus, even without PPI therapy, chyme entering the duodenum has a pH that is nearly neutral.

Higher dietary protein intake with a subsequently higher dietary acid load seems to increase calcium absorption compared with calcium absorption during a low-protein diet.(40,41) Thus, if our subjects consumed a high-protein diet or had a high dietary acid load, we might be unable to detect a PPI-mediated decrease in FCA. However, our subjects consumed a low potential renal acid load, and their nutritional habits were replicated during each study visit. Thus we feel that the acidity of subjects' meals had no significant impact on our study findings.

Three prospective studies, each designed to identify risk factors for osteoporotic fracture, documented that PPI therapy is an independent risk factor for osteoporotic fracture in older adults.(5,6) However, our study suggests that in postmenopausal women this relationship is not due to a direct negative effect on calcium absorption. We hypothesize that PPIs might adversely affect skeletal health through a different, and possibly indirect, mechanism that might be evident only following prolonged use.

PPIs might increase the risk of osteoporotic fracture by several mechanisms. PPI use can cause vitamin B_12_ deficiency,(42) which might indirectly increase the risk of fracture by causing peripheral neuropathy and increased falls or by causing high homocysteine levels with adverse effects on cross-linking of bone collagen.(43–47) Additionally, PPI therapy might decrease absorption of protein,(48) with potential long-term adverse effects on bone health. Bacterial overgrowth also can complicate prolonged PPI therapy(30,49) and might alter absorption of nutrients, contributing to fracture risk.

In conclusion, 30 days of continuous PPI therapy did not alter FCA in our cohort. Future studies should focus on identifying the mechanisms by which PPIs increase the risk of osteoporotic fracture to confirm a causal relationship. The underlying gastrointestinal condition for which acid suppression is prescribed might be the reason for a higher risk of osteoporotic fracture rather than the treatment itself. Additionally, comorbid diseases might explain the association between PPI therapy and risk of fracture. In a survey of 168,727 adults,(7) approximately 3% received long-term acid-suppression therapy. Patients taking acid suppressants had more medical illnesses and sought medical care significantly more often than patients not taking acid suppressants.(7) Thus PPI therapy simply may mark individuals who, owing to poor health, have a higher risk of osteoporotic fracture.
